# Comparison of Digital Rectal Thermometry and a Non-Contact Veterinary Infrared Thermometer in Cats: Identifying Alternative Sites to Rectal Measurement

**DOI:** 10.3390/vetsci12070618

**Published:** 2025-06-25

**Authors:** Carlotta Tombolani, Daniela Alberghina, Mauro Gioè, Fausto Quintavalla

**Affiliations:** 1 Indipendet Veterinary Practitioner, 37100 Verona, Italy; 2 Department of Veterinary Science, University of Messina, Via Palatucci 13, 98168 Messina, Italy; 3 Independent Researcher, Altofonte, 90072 Palermo, Italy; 4 Department of Veterinary Science, University of Parma, Via del Taglio 10, 43126 Parma, Italy

**Keywords:** thermometer, cat, body temperature, eye, perineal region

## Abstract

Background: Rectal temperature measurement in cats, while crucial, can cause discomfort and stress. This study evaluated non-contact infrared thermometry as a less invasive alternative. Methods: A total of 95 cats were enrolled in this study. The cats were categorized into three age groups: Group I (n = 20 kittens, 2–6 months), Group II (n = 34 young cats, 7–24 months), and Group III (n = 41 adult cats, >24 months). Results: The mean rectal temperature in cats was approximately 38 °C, which was significantly higher than both ocular temperature (*p* < 0.0001) and auricular pinna temperature (*p* < 0001). No statistically significant difference was found between rectal and perineal temperatures, nor in body temperatures between the age groups. Ocular temperature (*p* < 0.05) and auricular temperature (*p* < 0.0001) were influenced by ambient temperature. Perineal infrared temperatures showed a strong correlation and low bias compared to rectal temperature and were not affected by ambient temperature. Conclusions: Non-contact infrared thermometry offers advantages for feline temperature monitoring. Perineal infrared temperatures appear to be a useful, non-invasive alternative to rectal measurements in cats.

## Simple Summary

Using body temperature to assess health and identify infectious diseases in cats can sometimes be tricky for vets. This is because the most common method, measuring rectal temperature, can be difficult. This led us to seek a less invasive, more hygienic tool that prioritizes animal welfare. Our research aimed to validate a new veterinary non-contact infrared thermometer for use in cats. We took thermal measurements at the eye, ear pinna, and perineal area, and then compared these readings to rectal temperatures taken with a digital thermometer. Our various measurements showed that the instrument we used is effective for detecting body temperature in the perineal region.

## 1. Introduction

Body temperature is a reliable indicator of an animal’s physiological and pathological state. Its accurate measurement is crucial during a physical examination to inform treatment decisions and optimize patient care. Precise temperature determination is essential for assessing animal well-being, diagnosing febrile states (e.g., infections, immune-mediated diseases, endocrine disorders, systemic inflammatory response) in dogs (*Canis familiaris*) and cats (*Felis catus*), and identifying opposite clinical situations characterized by hypothermia [1].

Rectal temperature measurement, typically performed using digital thermometers, is widely considered the gold standard for obtaining core body temperature in pets [2]. Digital thermometers can be categorized into two main types: digital equilibrium thermometers, which require approximately 45 s to equilibrate with the temperature of the adjacent mucosa, and digital predictive thermometers, which analyze the initial rate of temperature change and employ mathematical algorithms to forecast the final temperature within 10–15 s [3].

Despite its accuracy, rectal temperature measurement presents significant practical and welfare challenges, particularly in cats. Rectal temperature measurement in cats is often challenging due to the procedure’s discomfort and stress, making restraint difficult for many animals. It can also be impossible in aggressive cats or those with anal lesions. The necessary restraint can induce stress, potentially leading to a transient elevation in body temperature, thereby impacting the accuracy of the reading. Furthermore, while devices with flexible tips can reduce the risk of intestinal mucosa perforation, the procedure still carries a minimal risk of injury [4]. These drawbacks, coupled with the potential for cross-contamination, highlight the urgent need for alternative, more comfortable, and equally reliable methods for measuring body temperature in pets [3]. For optimal animal welfare, an alternative temperature measurement method must be both accurate and minimally stressful [5].

More recently, alternative methods for body temperature detection have emerged, including auricular thermometry using infrared technology. However, studies have shown that auricular infrared thermometry can underestimate body temperature in dogs [6] and prove difficult to perform, especially in pets with otitis externa [7]. The advent of non-contact infrared thermometers and microchip temperature transponders offers non-invasive methodologies for measuring surface body temperature, thereby eliminating the necessity for physical or chemical restraint [8]. However, microchip use has been linked to potential complications such as granulomatous inflammation, spinal cord injuries from incorrect placement [9,10], and even the development of neoplastic processes in pets [11,12]. Given the limitations of existing methods, particularly for feline patients, and the critical importance of accurate temperature assessment in veterinary practice, there is a clear and unmet need for a non-invasive, reliable, and welfare-friendly method of temperature determination in cats. While previous research has explored various alternatives, none have fully addressed the combined requirements of accuracy, ease of use, and minimal stress in the unique feline context. This study directly addresses this gap by comprehensively investigating the correlation between the gold-standard digital rectal temperature and infrared temperatures obtained from three novel, less-explored anatomical sites in cats: ocular, inner ear pinna, and perineal locations. By systematically evaluating these specific sites, we aim to identify a practical and accurate alternative that prioritizes feline well-being, reduces stress for both animals and clinicians, and has the potential to become a valuable tool in routine veterinary examinations and emergency settings. This approach seeks to provide data that will directly inform clinical decision making, ultimately enhancing patient care and welfare in cats.

## 2. Materials and Methods

The University of Parma’s Animal Ethics Committee approved this study (04/CESA/2025), and all procedures adhered to relevant Italian guidelines and regulations.

In the present study, a total of 95 cats, ranging from 2 to 216 months of age (51 females and 44 males, mean age 43.97 months), were clinically examined. The subjects were all European breed cats, except for 3 Maine Coons, 3 Persians, 2 Birmans, 2 Siamese, 2 Scottish Folds, 1 Chartreux, and 1 Sphynx. The cats were categorized into three age groups: Group I (n = 20 kittens, 2–6 months, mean 5.25 months), Group II (n = 34 young cats, 7–24 months, mean 15.53 months), and Group III (n = 41 adult cats >24 months, mean 87.22 months). All animals were privately owned, and measurements were conducted at a veterinary clinic during general check-ups, vaccine boosters, and before sterilization procedures. At the time of the evaluations, the animals showed no visible clinical signs of disease. The study occurred in Italy, in the province of Verona, between October 2024 and April 2025.

## 3. Data Collection

In all examined animals, rectal temperature was recorded as the gold standard using a Vedo-Family Pic Solution digital thermometer with a rigid probe (Pikdare S.p.a., Casnate con Bernate, CO, Italy). The measurement took approximately 60 s, with the probe inserted into the rectum to a depth of about 12–15 mm, in contact with the dorsal rectal wall. Before each measurement, the instrument was routinely disinfected with an alcohol-soaked cotton pad.

**Figure 1:**
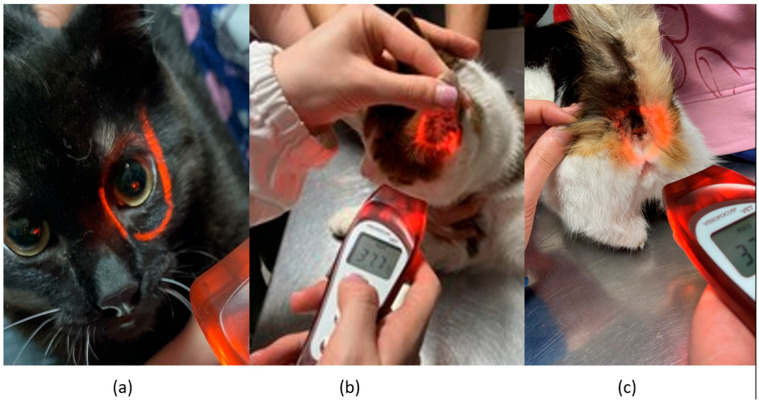
This figure represents the sites of temperature measurements detected in the cat: (**a**) central eye region; (**b**) inner ear; (**c**) perineum region.

Infrared temperature readings were taken immediately following the rectal measurements. A single, commercially available non-contact infrared thermometer (Visiofocus Vet, Tecnimed srl, P.le Cocchi, Vedano Olona, VA, Italy), previously validated in horses [13], was used. The infrared thermometer was used in accordance with the manufacturer’s instructions. The infrared thermometer was stabilized by allowing it to equilibrate with the ambient temperature, which was measured by the device itself on a wall. We used the Manual Quick Calibration System for this process. The device is equipped with an optical positioning system that projects four convergent light beams forming a circle on the area of interest, ensuring the correct target distance for accurate temperature detection. Infrared temperature readings were taken at the distance automatically indicated by the device from the central cornea region of the eye, the inner ear pinna toward the tympanic cavity, and the anal triangle of the perineum in cats (Figure 1). At each site, temperature readings were performed twice, with a 30 s interval between each reading.

## 4. Data Analysis

Statistical analysis was performed using GraphPad Prism version 8.4.2 (GraphPad Software, La Jolla, San Diego, CA, USA). The Shapiro–Wilk test was used to assess data normality, which revealed that not all data were normally distributed. To determine if there were significant differences in temperatures among the groups, a one-way ANOVA was performed, followed by Dunnett’s multiple comparisons test for specific group differences. Infrared temperatures from different body sites were then compared with digital rectal temperatures using a Mann–Whitney U test. Bland–Altman analysis was employed to assess the agreement between rectal and infrared temperatures. Pearson’s correlation coefficients were also calculated to determine the relationship between digital and infrared temperatures at different measurement locations. Given that correlations alone are statistically insufficient as a replacement for rectal temperature due to the influence of ambient temperature, a gamma generalized linear model (GLM) in R (version 3.5.0, R Foundation for Statistical Computing) was used to analyze the relationship between ambient and body temperatures. In all analyses, a *p*-value less than 0.05 was considered statistically significant.

## 5. Results

**Figure 2:**
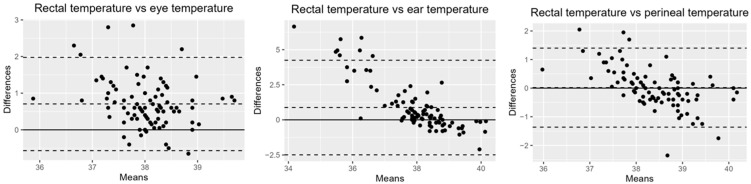
Bland–Altman plot of agreement between rectal temperature measurements and non-contact infrared temperature readings. The solid line marks the zero point, and the dashed lines show the bias (the central line) along with the Limits of Agreement (LOA), which are the lines at the extremes.

The mean ambient temperature recorded during data collection was 22.14 ± 1.56 °C. As shown in Table 1, the mean rectal temperature in cats was 38.42 ± 0.64 °C, which was significantly higher than the ocular temperature (U = 2068, *p* < 0.0001) and inner ear pinna temperature (U = 3064, *p* < 0.0001). Statistically, no significant difference was found between rectal and perineal temperatures, and similarly, no significant difference was observed in body temperatures between age groups. Bland–Altman analysis revealed that the perineum had the smallest bias relative to rectal temperature: 0.02 (95% Confidence Interval [CI]: −0.12, 0.16; Limit of Agreement [LOA]: −1.36, 1.40). This was followed by the eye: 0.71 (95% CI: −0.57, 0.84; LOA: −0.57,1.98). The bias for the inner ear pinna was notably higher: 0.88 (95% CI: 0.53, 1.23; LOA: −2.49, 4.25) (Figure 2).

**Figure 3:**
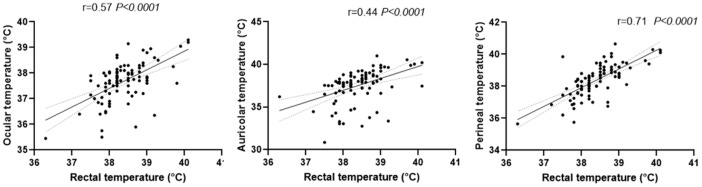
Correlation analysis of rectal and infrared temperatures for each body site in the studied feline population. The solid line represents the correlation coefficient, whereas the dashed lines denote the confidence intervals.

In addition, Figure 3 shows significant positive correlations between digital rectal temperature and infrared temperatures measured at the ocular, auricular, and, most notably, perineal sites. Gamma regression analyses were used to examine the relationship between ambient and body temperatures. Table 2 details significant relationships identified between rectal temperature and infrared temperatures at both the eye (*p* < 0.0001) and perineum (*p* < 0.0001). Although Pearson analysis revealed a correlation between rectal temperature and inner ear pinna temperature, this finding is likely confounded by the significant relationship observed in our gamma regression analysis between inner ear pinna temperature and ambient temperature (*p* < 0.0001).

## 6. Discussion

The mean rectal temperature observed in this study in cats was 38.40 °C (SD = 0.60 °C), which falls within the physiological range (36.7–38.9 °C/98.1–102.1 °F) previously reported for digital rectal temperatures of 200 healthy adult cats measured indoors under climate-controlled conditions [14]. In the present study, we used cats of all ages and found no significant differences between the groups for either digital rectal temperature or infrared temperature across the various measurement sites.

In human medicine, temperature detection with non-contact infrared instruments has proven to be particularly useful in pediatric patients since they do not always cooperate with rectal or oral devices [15]. This is where the idea of extending the application to veterinary medicine was born; however, the use of non-contact infrared thermometers does not always appear to provide reproducible or consistent data [16]. While rectal temperature is considered the standard measurement to assess body temperature in conscious animals, it can be stressful to perform. Our study aimed to assess how well infrared temperatures from the ocular, inner ear pinna, and perineal regions correlate with and agree with rectal temperature in cats. We found that infrared temperatures at the ocular and inner ear pinna sites were significantly lower than rectal temperatures, a finding consistent with prior research comparing rectal and non-contact infrared measurements at various body locations in cats [15,17]. This aligns with Lima et al.’s [18] observation that tympanic infrared thermometry tends to yield lower temperatures than rectal measurements, particularly in cats with normal or high body temperatures. Furthermore, although Pearson analysis revealed a correlation between rectal temperature and inner ear pinna temperature, this finding is likely confounded by the significant relationship observed in our gamma regression analysis between inner ear pinna temperature and ambient temperature. Consequently, ear pinna temperature measurement is not recommended as a reliable alternative to rectal measurement based on these results. The ocular region has been identified as a reliable thermal window in cats, demonstrating high sensitivity and specificity [8,19]. Notably, we observed a good relationship between rectal and ocular infrared temperatures in our study, which differs from the findings of Nutt et al. [15] and Giannetto et al. [16] who used different thermometric instruments in cats. This discrepancy suggests that the non-contact infrared thermometer we employed may offer improved performance. Unfortunately, ocular temperature is related to ambient temperature and this is a limitation of its use.

There was good agreement and a strong relationship between infrared perineal temperature and rectal temperature in cats, which is in agreement with what has recently been reported by other authors [20]. We found similar results in equines [13]. One advantage of infrared thermometry over rectal thermometry is the shorter pet handling and procedure time. Efforts to minimize stress in the veterinary clinic are an active area of research [21]. Patients who have had negative experiences are more fearful in subsequent veterinary appointments, and prolonged stress can adversely affect patient health [21].

A limitation of this study is the fixed order of temperature measurement, where rectal temperature was consistently obtained before infrared thermography. While this approach aimed to establish a gold-standard baseline before a less invasive method, it is acknowledged that the rectal procedure itself can induce a stress response in cats, potentially leading to a transient elevation in core body temperature. Although our infrared thermography readings remained consistently lower than rectal temperatures, suggesting this effect did not artificially narrow the observed difference, the potential for a stress-induced elevation of the initial rectal temperature cannot be entirely excluded. Future studies might consider crossover designs or the randomization of measurement order, where feasible, to more comprehensively assess potential order effects. However, practical constraints with animal welfare and maintaining a minimal handling approach remain a challenge in such experimental setups. Another limitation of this study was the lack of stress level evaluation in cats. A recent study found that assessed stress levels were almost twice as high when cats had their temperature measured rectally compared to in their ears [22]. Further research should investigate potential variations in perineal infrared temperature using non-contact infrared veterinary thermometers across diverse management settings in cats.

## 7. Conclusions

Non-contact veterinary infrared thermometry offers advantages for monitoring temperature in cats. This study found that perineal infrared temperatures showed a strong correlation and low bias compared to rectal temperature and were not affected by ambient temperature.

## Figures and Tables

**Table 1:** Mean ± standard deviations of rectal and infrared temperatures in the studied feline population across different age groups. Data include digital rectal, infrared, and ambient temperatures for Group I (Kittens: 2–6 months), Group II (Young cats: 7–24 months), and Group III (Adult cats: >24 months).

Body Site	Totals (95)	Group I (20)	Group II (34)	Group III (41)	ANOVA
Rectal (°C)	38.40 ± 0.60	38.29 ± 0.43	38.25 ± 0.45	38.56 ± 0.70	NS
Ocular (°C)	37.69 ± 0.76 ^a^	37.76 ± 0.54	37.70 ± 0.79	37.62 ± 0.84	NS
Auricolar (°C)	37.52 ± 1.90 ^a^	37.47 ± 2.03	37.13 ± 1.99	37.76 ± 1.83	NS
Perineal (°C)	38.38 ± 0.99	38.58 ± 0.66	38.12 ± 0.96	38.44 ± 1.08	NS
**Ambient**					
Temperature °C	22.14 ± 1.56	22.65 ± 2.01	21.99 ± 1.26	21.90 ± 1.52	NS

Vs rectal temperature: Mann–Whitney U test ^a^ *p* < 0.0001.

**Table 2:** The relationship between ambient and body temperatures was investigated using gamma regression analyses. In this model, infrared temperatures served as the response variables, while rectal and ambient temperatures were the explanatory variables.

	Explanatory Variables	
**Response variables**	Rectal Temperature	Ambient temperature
Eye temperature	*p* < 0.0001	*p* < 0.05
Inner ear pinna	*p* < 0.05	*p* < 0.0001
Perineal temperature	*p* < 0.0001	*p* = 0.83

## Data Availability

Links to publicly archived datasets analyzed: dx.doi.org/10.6084/m9.figshare.29383442.

## References

[1] Oncken A., Kirby R., Rudloff E. (2001). Hypothermia in critically ill dogs and cats. Compend. Contin. Educ. Pract. Vet..

[2] Konietschke U., Kruse B.D., Müller R., Stockhaus C., Hartmann K., Wehner A. (2014). Comparison of auricular and rectal temperature measurement in normothermic, hypothermic, and hyperthermic dogs. Tierarztl. Prax. Ausg. K Kleintiere Heimtiere.

[3] Kreissl H., Neiger R. (2015). Measurement of body temperature in 300 dogs with a novel noncontact infrared thermometer on the cornea in comparison to a standard rectal digital thermometer. J. Vet. Emerg. Crit. Care.

[4] Rexroat J., Benish K., Fraden J. (1999). Clinical Accuracy of Vet-Instant Ear Thermometer.

[5] Sousa M.G. (2016). Measuring body temperature: How do different sites compare?. Vet. Rec..

[6] Southward E., Mann F.A., Dodam J., Wagner-Mann C.C. (2006). A comparison of auricular, rectal and pulmonary artery thermometry in dogs with anesthesia induced hypothermia. J. Vet. Emerg. Crit. Care.

[7] Gonzalez A.M., Mann F.A., Preziosi D.E., Meadows R.L., Wagner-Mann C.C. (2002). Measurement of body temperature by use of auricular thermometers versus rectal thermometers in dogs with otitis externa. J. Am. Vet. Med. Assoc..

[8] Casas-Alvarado A., Mota-Rojas D., Hernández-Ávalos I., Mora-Medina P., Olmos-Hernández A., Verduzco-Mendoza A., Reyes-Sotelo B., Martínez-Burnes J. (2020). Advances in infrared thermography: Surgical aspects, vascular changes, and pain monitoring in veterinary medicine. J. Therm. Biol..

[9] Joslyn S.K., Witte P.G., Scott H.W. (2010). Delayed spinal cord injury following microchip placement in a dog. Vet. Comp. Orthop. Traumatol..

[10] Schneider N., Blutke A., Parzefall B. (2022). Recovery after inadvertent intramedullary microchip implantation at C1–C2 in a kitten. J. Feline Med. Surg. Open Rep..

[11] Vascellari M., Melchiotti E., Mutinelli F. (2006). Fibrosarcoma with typical features of postinjection sarcoma at site of microchip implant in a dog: Histologic and immunohistochemical study. Vet. Pathol..

[12] Carminato A., Vascellari M., Marchioro W., Melchiotti E., Mutinelli F. (2011). Microchip-associated fibrosarcoma in a cat. Vet. Dermatol..

[13] Alberghina D., Tombolani C., Quintavalla F. (2025). Performance of a non-contact veterinary infrared thermometer and reference intervals of equine temperature at different body sites. Front. Vet. Sci..

[14] Levy J.K., Nutt K.R., Tucker S.J. (2015). Reference interval for rectal temperature in healthy confined adult cats. J. Feline Med. Surg..

[15] Nutt K.R., Levy J.K., Tucker S.J. (2015). A Comparison of non-contact infrared thermometry and rectal thermometry in cats. J. Feline Med. Surg..

[16] Giannetto C., Acri G., Pennisi M., Piccione G., Arfuso F., Falcone A., Giudice E., Di Pietro S. (2022). Short Communication: Use of Infrared Thermometers for Cutaneous Temperature Recording: Agreement with the Rectal Temperature in *Felis catus*. Animals.

[17] Barton J.C., Didier M.D., Silvestrini P., German A.J., Ferriani R. (2022). A noninvasive method of temperature measurement using a noncontact handheld infrared thermometer fails to correlate with rectal temperature in dogs and cats. J. Am. Vet. Med. Assoc..

[18] Lima B.A., Ferreira L.C., Vilela V.L.R., Feitosa T.F. (2021). Temperature measurements in cats using digital, mercury and tympanic infrared thermometers. Ars Vet..

[19] Giannetto C., Di Pietro S., Falcone A., Pennisi M., Giudice E., Piccione G., Acri G. (2021). Thermographic ocular temperature correlated with rectal temperature in cats. J. Therm. Biol..

[20] Naimon N., Jarudecha T., Sussadee M., Muikaew R., Charoensin S. (2024). Prediction model for rectal temperature in cats with different baseline characteristics using a non-contact infrared thermometer. Vet. World.

[21] Scalia B., Alberghina D., Panzera M. (2017). Influence of Low Stress Handling during Clinical Visit on Physiological and Behavioural Indicators in Adult Dogs: A Preliminary Study. Pet Behav. Sci..

[22] Hanström Y., Oltegen S., Eklund I., Gröndahl E., Liszke I., Söder J. (2025). Assessed Temperatures and Stress in Cats Using Tympanic and Rectal Thermometers. Vet. Sci..

